# Advancing biliary tract malignancy treatment: emerging frontiers in cell-based therapies

**DOI:** 10.3389/fimmu.2025.1559465

**Published:** 2025-02-12

**Authors:** Jianyang Ao, Mingtai Hu, Jinghan Wang, Xiaoqing Jiang

**Affiliations:** Institute of Hepatobiliary and Pancreatic Surgery, Department of Hepatobiliary and Pancreatic Surgery, Shanghai East Hospital, School of Medicine, Tongji University, Shanghai, China

**Keywords:** biliary tract malignancies, cell therapy, immunotherapy, tumor microenvironment, combined therapy

## Abstract

Biliary tract malignancies, including intrahepatic cholangiocarcinoma, extrahepatic cholangiocarcinoma, and gallbladder cancer, represent a group of aggressive cancers with poor prognosis due to late-stage diagnosis, limited treatment options, and resistance to conventional therapies like chemotherapy and radiotherapy. These challenges emphasize the urgent need for innovative therapeutic approaches. In recent years, cell-based therapies have emerged as a promising avenue, offering potential solutions through immune modulation, genetic engineering, and targeted intervention in the tumor microenvironment. This Mini-review provides an overview of current advancements in cell-based therapies for biliary malignancies, encompassing immune cell-based strategies such as CAR-T cells, NK cells, dendritic cell vaccines, and tumor-infiltrating lymphocytes. We also examine strategies to overcome the immunosuppressive tumor microenvironment and discuss the integration of cell therapies into multimodal treatment regimens. By synthesizing preclinical and clinical findings, this review highlights key insights and future directions, aiming to assist researchers and clinicians in translating these approaches into effective treatments. The transformative potential of cell-based therapies discussed here makes this review a valuable resource for advancing biliary malignancy research and clinical applications.

## 1 Introduction

Biliary tract malignancies, including intrahepatic cholangiocarcinoma(ICC), extrahepatic cholangiocarcinoma(ECC), and gallbladder cancer (GBC), rank among the most lethal cancers, with dismal five-year survival rates and limited progress in improving outcomes over the past decades (1–3). The incidence of biliary tract cancer varies by region, with cholangiocarcinoma having a low incidence in high-income countries (0.35 to 2 cases per 100,000 annually) and much higher rates in endemic areas like Thailand and China, where it can be up to 40 times higher. For patients with advanced biliary tract cancer, survival remains poor, with median overall survival ranging from 2.5 to 4.5 months, as shown in randomized controlled trials (1). Biliary tract malignancies often present asymptomatically in early stages, leading to late-stage diagnoses where curative surgical options are no longer feasible. Moreover, biliary tumors are inherently resistant to conventional therapies, including chemotherapy and radiotherapy, which are further hampered by the tumor’s dense stromal barrier and immunosuppressive microenvironment (4). The standard treatment regimens for biliary malignancies, including Gemcitabine-based chemotherapy or Cisplatin and Gemcitabine combinations, provide only modest survival benefits and are frequently associated with significant toxicity (5). Targeted therapies aimed at genetic alterations such as FGFR2 fusions, IDH1 mutations (6), and HER2 overexpression have shown promise in a subset of patients but remain far from curative (7–9). Immunotherapy, a breakthrough in treating other cancers, has faced significant challenges in biliary malignancies due to the highly immunosuppressive tumor microenvironment (TME) (10, 11), characterized by regulatory T cells (Tregs), myeloid-derived suppressor cells (MDSCs), and cancer-associated fibroblasts (CAFs).

Cell-based therapies have emerged as a revolutionary approach, leveraging the body’s immune system or engineered cellular systems to combat malignancies (12, 13). Unlike conventional treatments, these therapies are designed to specifically target tumor cells, potentially overcoming the limitations of the TME and achieving durable responses. Among the most promising strategies are chimeric antigen receptor (CAR)-T cells, which have demonstrated success in hematological cancers and are now being adapted for solid tumors like biliary malignancies (14, 15). Natural killer (NK) cell therapies and dendritic cell (DC)-based vaccines are also being explored for their potential to activate innate and adaptive immune responses against these tumors (16, 17). Tumor-infiltrating lymphocytes (TILs), another emerging avenue, exploit the natural immune infiltration of tumors to enhance therapeutic outcomes (18).

Recent advancements in genetic engineering and synthetic biology, have significantly improved the precision and efficacy of cell-based therapies (19). These technologies allow for the modification of immune cells to enhance their tumor-targeting capabilities while minimizing off-target effects. Additionally, efforts to modulate the TME—such as targeting desmoplasia, reprogramming stromal components, and reducing immunosuppressive cytokines—are creating a more favorable environment for therapeutic cells to function effectively (20). This review explores the rapidly evolving landscape of cell-based therapies for biliary malignancies. By synthesizing insights from preclinical studies and clinical trials, we aim to highlight key challenges and opportunities, providing a comprehensive perspective on how cell-based strategies may redefine treatment paradigms for these challenging cancers.

## 2 Immune cell-based therapies

### 2.1 CAR-T cell therapy

**Figure 1:**
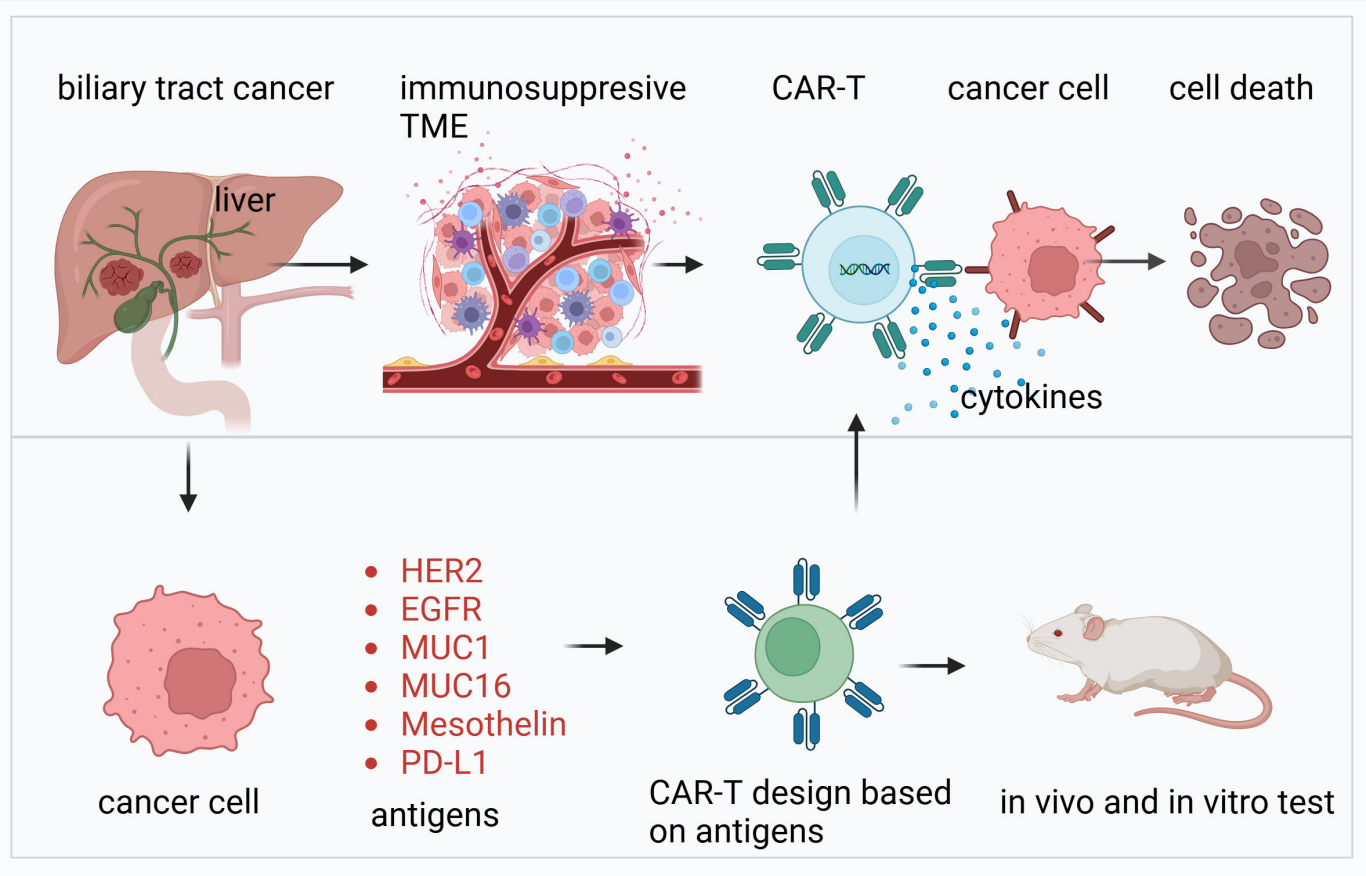
Overview of CAR-T cell therapy targeting biliary tract cancer. This figure illustrates the development and application of CAR-T cell therapy for biliary tract cancer. The upper panel highlights the immunosuppressive TME characteristic of biliary tract cancers, which hinders immune response. CAR-T cells, engineered with antigen-specific receptors, are shown to recognize and bind to cancer cells, leading to the release of cytokines and subsequent tumor cell death. The lower panel details the design and validation process of CAR-T cells. Antigens such as HER2, EGFR, MUC1, MUC16, mesothelin, and PD-L1, commonly overexpressed in biliary tract cancers, are selected as targets for CAR-T design. These CAR-T cells are tested for efficacy and safety through in vitro and in vivo experiments, including animal models, to ensure their therapeutic potential.

CAR-T cell therapy has emerged as a promising immunotherapeutic strategy for biliary malignancies. By engineering T cells to express CARs that specifically target tumor-associated antigens, CAR-T cells overcome the limitations of conventional therapies. In biliary cancers, targets such as mesothelin, HER2, and EGFR have been identified as promising candidates for CAR-T cell therapy. Mesothelin, frequently overexpressed in biliary cancers, is implicated in tumor progression through its role in cell adhesion, migration, and immune evasion (21). By interacting with MUC16, mesothelin promotes metastasis and suppresses immune responses, contributing to therapy resistance. Overexpression of mesothelin predicts malignant progression of cholangiocarcinoma (22). CAR-T cells targeting mesothelin have shown potential in reducing tumor burden, though their success has been hindered by the highly immunosuppressive TME and off-target toxicity. Mechanistically, mesothelin CAR-T cells secreting anti-CD3 molecules efficiently targeting pancreatic cancer and ovarian cancer (23, 24). The affinity-tuned mesothelin CAR-T cells indicate the potentiated targeting specificity and reduced off-tumor toxicity (25). Moreover, gallbladder cancer tumors frequently express carcinoembryonic antigen (CEA),CEA-specific CAR-T cells effectively recognize and respond to CEA, even in the presence of immune-suppressive factors like PD-L1. CAR-T cells showed strong activation, cytokine production (IFN-γ, TNF-α), and cytotoxicity, reducing tumor growth *in vitro* and *in vivo* in gallbladder cancer (26). In addition, chronic cholangitis is a significant risk factor for cholangiocarcinoma due to prolonged inflammation and bile duct damage. PD-1-targeting CAR-T cells effectively treat autoimmune cholangitis by selectively depleting pathogenic CD8^+^ tissue-resident memory T (Trm) cells in the liver, which alleviates biliary immunopathology and highlights the therapeutic potential of CAR-T cell therapy biliary cholangitis (27). Recent research has indicated that EGFR and B7H3 antigens are highly expressed in biliary tract cancer, EGFR-CAR-T and B7H3-CAR-T cells demonstrated specific anti-tumor activity (28). Similarly, HER2, a receptor tyrosine kinase overexpressed in a subset of biliary cancers, drives oncogenic signaling through pathways like PI3K/AKT and MAPK (29). These pathways enhance tumor cell proliferation, survival, and resistance to therapies, including chemotherapy and immune checkpoint inhibitors. HER2 is overexpressed in a subset of biliary cancers, is being explored as a target, with clinical trials evaluating its therapeutic potential by pertuzumab and trastuzumab (30, 31), and Zanidatamab (32). HER2- targeting CAR-T cells has been successful in pre-clinical research in glioblastoma (33), gastric cancer (34), ovarian cancer (35), breast cancer (36), and in clinical trial for targeting sarcoma (37). Therefore, CAR-T cells targeting HER2 will probably have promising result for biliary malignancies (**Figure 1**). Currently, there is insufficient evidence to support the clinical use of CAR T-cell therapy for advanced cholangiocarcinoma (CCA). Further clinical trials are essential to establish its safety and efficacy for routine clinical application. Notably, the safety and effectiveness of MUC-1 CAR T cells are being evaluated in a Phase I/II clinical trial in China for patients with ICC (NCT03633773) (38).

Despite encouraging preclinical results, CAR-T cell therapy faces significant challenges in biliary malignancies. The dense stromal barrier, composed of CAFs and extracellular matrix components, impedes CAR-T cell infiltration. Additionally, the immunosuppressive TME, characterized by Tregs and MDSCs, further limits efficacy. Moreover, the high expression of immune checkpoint molecules like PD-L1 within the TME suppresses CAR-T cell function, contributing to treatment resistance (39). through knock down immune checkpoints and immunosuppressive molecular receptors. Strategies to overcome these barriers include combining CAR-T therapy with immune checkpoint inhibitors and modulating the TME using stromal-targeting agents or cytokines.

### 2.2 Natural killer cell therapy

NK cells, integral to the innate immune system, possess unique advantages over T cells in cancer therapy, including their ability to target tumor cells without prior antigen sensitization. NK cell-based therapies for biliary malignancies encompass allogeneic NK cells, cytokine-induced killer (CIK) cells, and engineered NK cells. Allogeneic NK cells derived from healthy donors offer an off-the-shelf therapeutic option. Preclinical models of biliary cancers have demonstrated their efficacy, particularly when combined with cytokines like IL-2 or IL-15 to enhance their cytotoxic activity. Engineered NK cells, modified to express CARs or other tumor-specific receptors, represent an exciting avenue for enhancing specificity and potency.

Targeting PD-1 in cholangiocarcinoma by nanovesicle-based immunotherapy has exhibited good result in mouse modal (40). The biliary epithelium presents antigens to activate NK cells and NK T cells (41). Checkpoint inhibitors have shown synergistic effects when combined with NK cell therapies. By alleviating inhibitory signals within the TME, these combinations restore NK cell functionality and improve therapeutic outcomes. Current clinical trials are exploring the integration of NK cell therapies with checkpoint inhibitors in biliary malignancies.

### 2.3 Dendritic cell -based vaccines

DCs, as potent antigen-presenting cells, play a pivotal role in initiating robust T cell responses (42). DC-based vaccines, designed to present tumor-specific antigens, have shown promise in biliary malignancies (17, 43). Personalized DC vaccines using neoantigens derived from individual tumors are at the forefront of this approach.

Clinical trials have demonstrated the safety and immunogenicity of DC-based vaccines in melanoma-associated antigen (MAGE)-positive gallbladder carcinoma (44). For example, vaccines loaded with peptides from commonly mutated genes, such as KRAS (45), or tumor lysates have induced tumor-specific immune responses. Combining DC vaccines with immune checkpoint blockade has further enhanced efficacy by enabling sustained T cell activation and memory formation.

The success of vaccination depends on both the immune system’s capabilities and the choice of an appropriate target antigen. Ideally, the target should be tumor-specific and conserved within the cancer cells to minimize collateral damage to healthy tissues and reduce the risk of antigen-negative tumor cells that might evade immune detection. Wilm’s Tumor protein 1 (WT1), a transcription factor that plays a role in urogenital development and tumor suppression, is a prime candidate for cancer immunotherapy (46). WT1 interacts with various growth factor receptors and regulatory proteins, such as PDGF-R, EGFR, c-MYC, and Bcl-2 (47). WT1 mutations are present in 68%-80% of biliary tract cancers, and while the clinical relevance in biliary tract cancer is still under investigation, similar mutations have been associated with poor prognosis in other cancers like testicular, breast, and head and neck cancers (48). Another promising antigen is Mucin 1 (MUC1) (46), a heavily glycosylated glycoprotein found on the surface of many tumor cells. MUC1 helps form a protective barrier around tumors, preventing the penetration of chemotherapy and immune cells. Overexpression of MUC1 is observed in 90% of gallbladder carcinoma cases and 59%-77% of cholangiocarcinoma cases, often correlating with more advanced disease and poor overall survival (49). The effectiveness of peptide-based vaccines can be limited by the tumor heterogeneity within cancers like biliary cancer. Although antigens such as WT1 and MUC1 are often overexpressed in biliary tumors, their distribution is not uniform. Some cancer cells may lack these antigens entirely, or express them at low levels. Additionally, individual variations in immune response, even among patients with similar HLA types, can affect the success of these vaccines. This is due, in part, to differences in the number of lymphocytes that are sensitive to these specific antigens. Approaches to improve efficacy include incorporating adjuvants like Toll-like receptor (TLR) agonists and leveraging advanced delivery platforms, such as nanoparticles, to enhance antigen presentation and immune activation.

### 2.4 Tumor-infiltrating lymphocytes

TILs represent a highly promising approach to harness the body’s natural immune response against biliary malignancies (18, 50). These immune cells, which naturally migrate into tumor tissues, can be isolated and expanded from patient tumor specimens, generating autologous T cell products capable of recognizing and targeting tumor-associated antigens. Preclinical research has demonstrated the potential of TIL therapy to mediate tumor regression in models of biliary tract cancer (51, 52). In these studies, TILs were shown to infiltrate the TME, attack cancer cells, and lead to significant tumor reduction (53, 54). These results underscore the promise of TIL-based therapies in targeting cancers that are traditionally resistant to standard treatments. The combination of TIL together with other therapies often include cytokines such as interleukin-2 (IL-2), which can enhance TIL persistence and function, or immune checkpoint inhibitors (e.g., anti-PD-1/PD-L1), which may reverse immune suppression within the TME and enhance TIL survival and effectiveness. The aim of these combination therapies is to improve the persistence and functionality of TILs in the immunosuppressive and hostile TME often found in biliary tract cancer.

One of the major challenges in TIL therapy for biliary tract cancer is the difficulty in obtaining sufficient TILs from tumor tissues (55). Biliary tumors often exhibit low immunogenicity, which means that fewer T cells are naturally infiltrating the tumor (56, 57). This, combined with the immunosuppressive environment present in the TME, limits the availability and effectiveness of TILs. As a result, strategies to improve TIL efficacy have focused on several key areas: (1) preconditioning regimens: These involve manipulating the TME before TIL infusion to reduce the presence of immunosuppressive cells such as Tregs and MDSCs. By depleting these cells, TILs are more likely to survive and function effectively. (2) Genetic modifications: enhancements such as chimeric antigen receptor (CAR) T cell therapy and TCR modifications have been used to increase TIL survival, improve their cytotoxic potential, and enhance their resistance to the hostile TME. CAR-T cells, for example, can be engineered to specifically target tumor antigens present on cancer cells (58). (3) Combination therapies: clinical trials exploring combinations of TIL therapy with cytokines and immune checkpoint inhibitors have shown promise in improving outcomes (59). These combinations aim to create a more favorable TME, enhancing TIL expansion, persistence, and tumor-killing capacity.

While TIL-based therapies for biliary tract cancer remain in their infancy, the ongoing research and pre-clinical research hold great potential for improving outcomes with this difficult-to-treat cancer. The ability to harness and enhance the immune system through TIL therapy may represent a major breakthrough in addressing the immune escape mechanisms that often render traditional therapies ineffective in biliary tract cancer. Continued advancements in understanding the biology of TILs, the challenges of the TME, and the development of personalized approaches will be essential to maximize the therapeutic potential of TILs in biliary cancers.

## 3 Other immunotherapies targeting biliary tract malignancies

### 3.1 Targeting immune checkpoints

Immune checkpoint expression plays a critical role in cholangiocarcinoma and is strongly associated with poor prognosis. PD-L1 is expressed in 49–94% of biliary tract cancer cases, and its presence correlates with worse survival outcomes, including a 60% reduction in survival for patients with high PD-L1 expression at the tumor front (60, 61). PD-1 and PD-L1 upregulation have been observed in biliary tract cancer subtypes, such as ECC and ICC, where they are linked to decreased CD8^+^ T-cell infiltration, poorer overall survival, and increased metastasis (62). Elevated soluble PD-L1 levels in the serum of advanced biliary cancer patients further predict worse survival. Other checkpoints, such as B7-H4 and FOXP3, are associated with poor prognosis, while CTLA-4 expression in certain biliary tract cancer subtypes has shown mixed outcomes, including improved disease-free intervals in hilar biliary tract cancer (63). Gene expression studies indicate that hypermutated biliary tract cancer tumors frequently exhibit increased checkpoint molecule expression, such as CTLA-4, PD-L1, and LAG3, actively suppressing the immune response (64, 65). These findings underscore the potential of targeting immune checkpoints, particularly PD-1/PD-L1, as a therapeutic strategy for biliary tract cancer, providing a foundation for ongoing clinical trials to evaluate their efficacy and safety.

### 3.2 Synthetic approaches

Synthetic biology has paved the way for designing immune cells with enhanced therapeutic capabilities. By incorporating synthetic genetic circuits, researchers create “smart” immune cells that precisely target tumors while minimizing off-target effects. One such innovation involves the use of AND-gate circuits, which require the presence of multiple tumor-associated antigens to activate the therapeutic response, thus improving specificity in targeting biliary tumors (66, 67). Safety switches represent another critical advancement in synthetic biology. These switches, often based on inducible suicide genes, allow clinicians to terminate cell therapy in case of severe adverse effects. For instance, synthetic circuits incorporating small-molecule-inducible caspase systems rapidly eliminate engineered cells in response to toxicity, ensuring patient safety (68).

Synthetic biology has also enabled the development of “armored” CAR-T cells, which are engineered to secrete cytokines such as IL-12 within the TME. These cytokines boost local immune activation and counteract immunosuppressive signals, amplifying the therapeutic impact on biliary malignancies. CAFs in the TME inhibit T cell infiltration and induce dysfunction, while the limited availability of tumor-specific antigens (TSAs) and expression of tumor-associated antigens (TAAs) on normal tissues can cause “on-target, off-tumor” toxicity. A TALEN-based strategy to engineer allogeneic “Smart CAR T cells” were developed (66). These cells express a CAR targeting FAP+ CAFs in solid tumors and a second CAR, directed at a TAA like mesothelin, integrated into a TCR signaling-inducible locus such as PDCD1 (66). A library of multi-receptor cell-cell recognition circuits was created using synthetic Notch receptors to link multiple molecular recognition events. These circuits enable engineered T cells to recognize both extracellular and intracellular antigens, showing robustness to heterogeneity and achieving precise recognition of up to three antigens with positive or negative logic (69). Additionally, synthetic biology approaches are being employed to design NK cells with enhanced migratory and cytotoxic properties, expanding their potential applications in treating solid tumors like biliary cancers (70).

Preclinical and early clinical studies are beginning to demonstrate the potential of combining ICIs with cell-based therapies in biliary tract cancer. For example, CAR-T cells targeting PD-L1 or other tumor-specific antigens could directly lyse tumor cells while benefiting from the additional immune activation provided by ICIs. Similarly, ICIs can enhance the persistence and function of adoptively transferred TILs or NK cells, improving their efficacy against biliary tract cancer. Despite these advances, challenges remain, including the heterogeneity of biliary cancers, the complexity of the TME, and potential toxicities from combining immune-based therapies. Strategies such as optimizing antigen selection for cell therapies, using novel immune checkpoint targets, and leveraging biomarkers to stratify patients will be crucial to maximizing the benefits of these combinations.

## 4 Modulating the tumor microenvironment in biliary tract cancers

Biliary malignancies are characterized by a unique and challenging TME that presents significant barriers to effective therapy. The TME of these tumors is composed of a dense extracellular matrix (ECM), stromal cells, and various immunosuppressive elements, which not only limit the delivery of therapeutic agents but also actively support tumor progression and immune evasion. TME reprogramming by the combination of chemotherapy and CTLA-4 immune checkpoint block enhances anti-PD-1 therapy (71).

### 4.1 Cancer-associated fibroblasts and stroma cells confer immuno-suppressive TME

A defining feature of biliary cancers is their pronounced desmoplastic reaction, marked by extensive fibrosis and the accumulation of CAFs (72). CAFs secrete ECM components, such as collagen, hyaluronan, and fibronectin, creating a physical barrier that restricts the infiltration of therapeutic immune cells, including CAR-T cells, NK cells, and TILs (73). Moreover, CAFs produce immunosuppressive cytokines like transforming growth factor-beta (TGF-β) and interleukin-6 (IL-6), which inhibit the activity of cytotoxic T cells and NK cells, while promoting the recruitment of Tregs and MDSCs (74, 75). CAF promoted cholangiocarcinoma growth by secreting hyaluronan synthase 2 instead of type 1 collagen (76). In addition, Stromal cells also contribute to metabolic reprogramming within the TME, depriving immune cells of vital nutrients like glucose and amino acids, while enriching the environment with immunosuppressive metabolites such as adenosine and lactate (77). These factors create a hostile microenvironment for immune cell therapies, necessitating strategies to target stromal components directly.

### 4.2 Approaches to reprogram the TME

#### 4.2.1 Stromal cell targeting

One promising approach involves directly targeting CAFs to disrupt their tumor-supportive roles. Agents such as fibroblast activation protein (FAP)-specific inhibitors have demonstrated preclinical efficacy in depleting CAF populations, resulting in reduced fibrosis and enhanced immune cell infiltration. Additionally, efforts are underway to reprogram CAFs into a tumor-restraining phenotype using agents like vitamin D analogs, which have the potential to convert activated CAFs into a quiescent state.

#### 4.2.2 Enzyme-based desmoplasia modulation

Another strategy focuses on enzymatic degradation of the ECM to improve immune cell penetration. Enzyme-based desmoplasia modulation aims to degrade or remodel the ECM, reduce stromal density, and enhance the accessibility of therapeutic agents. The following enzymes have shown potential in biliary tract tumor models: (1)Hyaluronidase: Hyaluronidase degrades hyaluronic acid, a major component of the ECM that contributes to elevated interstitial fluid pressure (78). By breaking down hyaluronic acid, hyaluronidase reduces tumor stiffness, improves vascular perfusion, and facilitates the delivery of chemotherapeutics and immune cells. PEGPH20, a pegylated form of hyaluronidase, has shown the ability to degrade hyaluronan, a major ECM component, thereby facilitating the delivery of immune and chemotherapeutic agents (79). In preclinical models of biliary cancers, PEGPH20 has been shown to synergize with CAR-T cell therapy, enhancing T cell infiltration and efficacy (80). More of its roles have been studied in pancreatic cancer (81). They also enhance the efficacy of gemcitabine and other systemic therapies in desmoplastic tumors (82). (2) Collagenase: Collagenase targets collagen, the most abundant structural protein in the ECM, thereby weakening the fibrotic barrier (83). Collagenase treatment has been shown to improve T cell infiltration in desmoplastic tumors, making it a promising adjunct to immunotherapies such as immune checkpoint inhibitors or CAR-T cells. (3) Matrix metalloproteinases (MMPs): MMPs are endogenous enzymes capable of degrading various ECM components. Recombinant MMPs or MMP-inducing strategies have been explored to modulate the TME and enhance the penetration of therapeutic agents. MMP2 induces COL1A1 synthesis by integrin alpha V to promote cholangiocarcinoma metastasis (84). A selective NOTCH1 inhibitor Crenigacestat reduces ICC progression by blocking VEGFA/DLL4/MMP13 axis (85). By breaking down the desmoplastic stroma, Enzyme-based modulation improve drug delivery, alleviate hypoxia, and promote immune cell infiltration. Additionally, combining enzyme therapy with chemotherapy or immunotherapy may achieve synergistic effects, enhancing overall efficacy. However, challenges remain. The nonspecific activity of enzymes leads to off-target effects and toxicity in normal tissues. Moreover, the dynamic nature of the TME means that stromal remodeling must be carefully timed and dosed to avoid adverse effects, such as tumor promotion due to excessive ECM degradation.

#### 4.2.3 Immunomodulatory agents

TGF-β inhibitors reverse T cell exhaustion and reprogram the TME by alleviating fibrosis and immune suppression, making these inhibitors especially relevant for cholangiocarcinoma and other biliary tract cancers. Indoleamine 2,3-dioxygenase(IDO) inhibitors, which block the enzyme responsible for depleting tryptophan and suppressing T cell activity, are being tested in combination with NK cell therapies to enhance immune effector functions in these tumors (86, 87). Additionally, targeting adenosine signaling using A2A receptor antagonists helps counteract the immunosuppressive effects of adenosine, a metabolite enriched in the hypoxic and nutrient-deprived TME of biliary tumors (88). These strategies address the unique challenges of biliary tract cancers, where immune evasion and stromal barriers limit the efficacy of current treatments, offering new hope for improving therapeutic outcomes through combination approaches.

### 4.3 Combining cell-based therapies with TME modulators

Integrating cell-based therapies with TME-modulating agents has demonstrated potential in overcoming the challenges presented by the TME in biliary malignancies. For example, the combination of PEGPH20 and immune checkpoint inhibition has shown enhanced tumor regression in preclinical models of colon cancer (89). Similarly, using CAF-targeting agents alongside NK cell therapy has improved NK cell infiltration and cytotoxicity (90). Innovative approaches involving dual-function engineered cells are also under investigation. For example, CAR-T cells engineered to secrete ECM-degrading enzymes or immunostimulatory cytokines could simultaneously target tumor cells and modulate the TME (91). Additionally, NK cells equipped with synthetic receptors targeting stromal components like FAP or integrins may selectively disrupt the tumor stroma while maintaining anti-tumor activity, offering a promising strategy for tackling the complex TME of biliary malignancies (92).

## 5 Conclusions and perspectives

**Figure 2:**
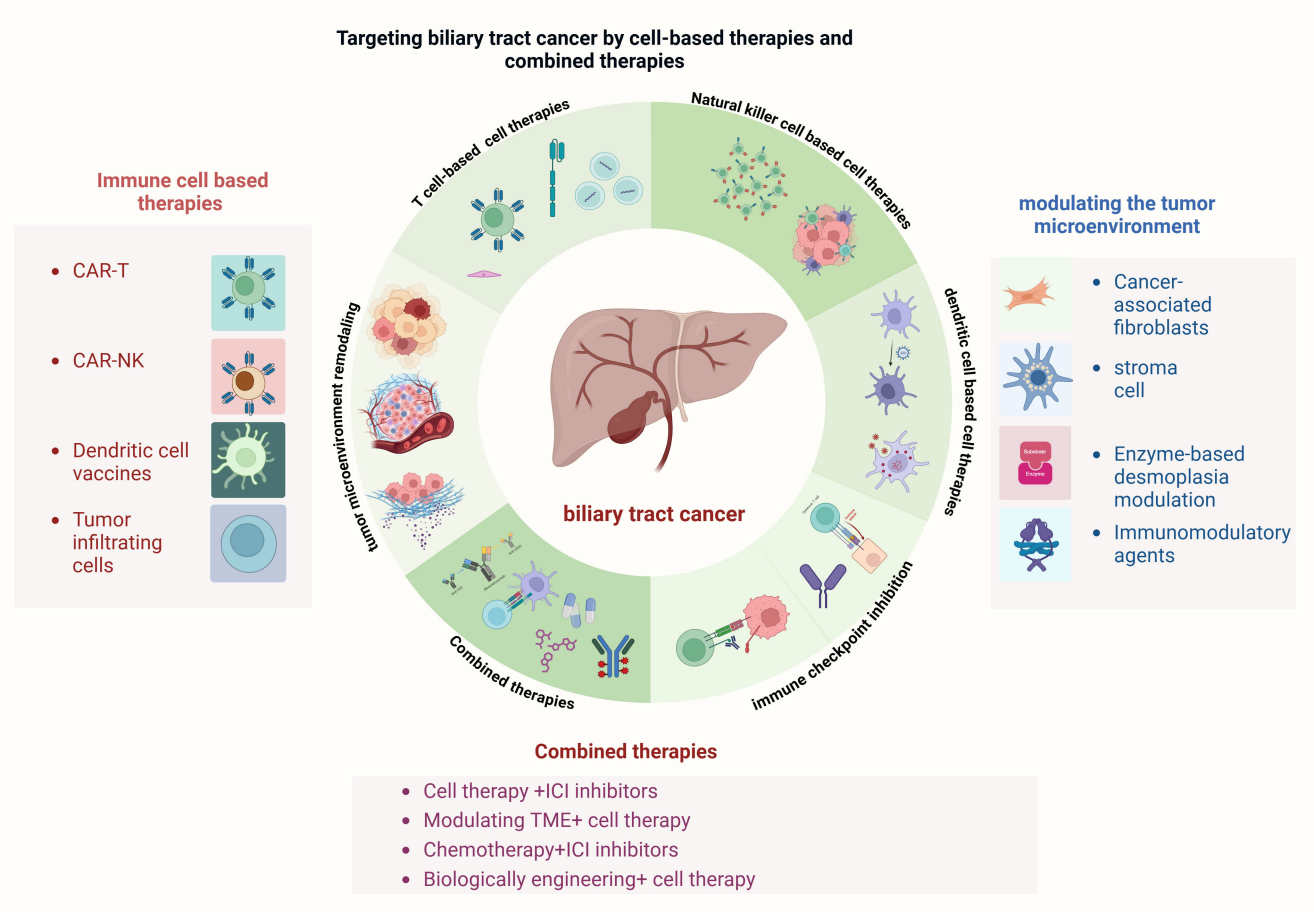
Targeting biliary tract cancer by cell-based therapies and combined therapies.

Recent advancements in cell-based therapies have introduced promising avenues for the treatment of biliary tract cancers. Immune cell-based strategies, including CAR-T cells, NK cells, and dendritic cell vaccines, alongside innovative genetic engineering techniques and synthetic biology, offer significant potential in addressing the challenges posed by these aggressive malignancies (**Figure 2**). However, the application of these therapies is still hindered by several critical challenges that must be addressed to improve their clinical utility. One major hurdle is the immunosuppressive TME in biliary tract cancers, which limits the efficacy of cell-based therapies by suppressing anti-tumor immune responses. Additionally, safety concerns such as cytokine release syndrome (CRS) and off-target effects present significant barriers to broader clinical application. CRS, a frequent complication in CAR-T therapy, results from excessive immune activation and can lead to life-threatening systemic inflammation. To address this, mitigation strategies such as the use of IL-6 receptor antagonists (e.g., tocilizumab), corticosteroids, and dose titration protocols have been developed. Off-target effects, caused by the unintended targeting of healthy tissues due to shared antigen expression, further complicate treatment. Advanced strategies, including dual-antigen targeting systems, tumor-restricted promoters, and engineered safety switches, are being explored to enhance therapeutic precision and minimize collateral damage. Furthermore, optimizing manufacturing processes and integrating emerging technologies, such as CRISPR-Cas9 and synthetic biology, are critical steps to enhance both the efficacy and safety of these therapies. Collectively, overcoming these challenges will be essential to unlocking the full potential of cell-based therapies and improving outcomes for patients with biliary malignancies. Current main open questions in targeting biliary tract cancer by cell therapy are as below:

- How to effectively overcome the immunosuppressive tumor microenvironment to enhance the efficacy of cell-based therapies for biliary tract cancers?
- What are the next critical steps in optimizing the manufacturing processes of cell-based therapies to ensure their scalability and accessibility for a broader patient population?
- In what ways can the integration of immune-modulating agents with cell-based therapies potentially synergize to improve treatment outcomes in biliary tract cancers?
- How might advancements in genetic engineering techniques and synthetic biology further enhance the precision and effectiveness of personalized cell therapies for individual patients with advanced biliary cancers?

The immune cell-based therapies section highlights approach such as CAR-T cells, CAR-NK cells, dendritic cell vaccines, and tumor-infiltrating lymphocytes, which aim to harness or engineer immune cells to combat cancer. The modulation of the tumor microenvironment includes targeting cancer-associated fibroblasts (CAFs), stromal cells, and using enzyme-based desmoplasia modulation or immunomodulatory agents to overcome the immunosuppressive environment. Combined therapies integrate cell therapies with immune checkpoint inhibitors (ICIs), chemotherapy, or biologically engineered therapies to enhance efficacy. These approaches aim to overcome the challenges of biliary tract cancer by improving immune recognition, tumor microenvironment remodeling, and therapeutic synergy.

The future of biliary tract cancer treatment lies in continued innovation and cross-disciplinary collaboration. By combining cell-based therapies with immune-modulating agents, advanced genetic tools, and personalized treatment strategies, there is a tangible potential to significantly improve outcomes for patients with biliary tract cancers. As research progresses, the clinical translation of these therapies will mark a transformative step in combating these challenging malignancies, offering renewed hope to patients who currently have limited effective treatment options.

To further advance biliary tract cancer treatment, several key research directions need to be explored. First, identifying and validating novel tumor-associated antigens is crucial. By leveraging high-throughput screening and bioinformatics approaches, researchers can uncover new targets for immunotherapy, potentially improving the specificity and efficacy of treatments. Second, enhancing delivery systems is essential to ensure the precise targeting of therapies to tumor sites. Developing advanced systems, such as nanoparticles or viral vectors, could significantly improve the efficiency and precision of cell-based treatments. Lastly, addressing the heterogeneity of TME is vital for overcoming resistance to therapies. The TME’s immunosuppressive nature hinders effective treatment; thus, targeting TME components like tumor-associated macrophages or regulatory T cells could help overcome these barriers and potentiate the effects of immunotherapies. By focusing on these research avenues, the therapeutic landscape for BTC can be dramatically improved, leading to more effective, personalized treatment options and better outcomes for patients.

## References

[1] Valle JW Kelley RK Nervi B Oh DY Zhu AX. Biliary tract cancer. Lancet. (2021) 397:428–44. doi: 10.1016/S0140-6736(21)00153-7 33516341

[2] Harding JJ Khalil DN Fabris L Abou-Alfa GK. Rational development of combination therapies for biliary tract cancers. J Hepatol. (2023) 78:217–28. doi: 10.1016/j.jhep.2022.09.004 PMC11111174 36150578

[3] Roa JC Garcia P Kapoor VK Maithel SK Javle M Koshiol J. Gallbladder cancer. Nat Rev Dis Primers. (2022) 8:69. doi: 10.1038/s41572-022-00398-y 36302789 PMC12314663

[4] Vogel A Segatto O Stenzinger A Saborowski A. FGFR2 inhibition in cholangiocarcinoma. Annu Rev Med. (2023) 74:293–306. doi: 10.1146/annurev-med-042921-024707 36170665

[5] Kelley RK Ueno M Yoo C Finn RS Furuse J Ren Z. Pembrolizumab in combination with gemcitabine and cisplatin compared with gemcitabine and cisplatin alone for patients with advanced biliary tract cancer (KEYNOTE-966): a randomised, double-blind, placebo-controlled, phase 3 trial. Lancet. (2023) 401:1853–65. doi: 10.1016/S0140-6736(23)00727-4 37075781

[6] Makawita S Lee S Kong E Kwong LN Abouelfetouh Z Danner De Armas A. Comprehensive immunogenomic profiling of IDH1-/2-altered cholangiocarcinoma. JCO Precis Oncol. (2024) 8:e2300544. doi: 10.1200/PO.23.00544 38547421 PMC10994443

[7] Lamarca A Barriuso J McNamara MG Valle JW. Molecular targeted therapies: Ready for “prime time” in biliary tract cancer. J Hepatol. (2020) 73:170–85. doi: 10.1016/j.jhep.2020.03.007 32171892

[8] Kam AE Masood A Shroff RT. Current and emerging therapies for advanced biliary tract cancers. Lancet Gastroenterol Hepatol. (2021) 6:956–69. doi: 10.1016/S2468-1253(21)00171-0 34626563

[9] Ho J Fiocco C Spencer K. Treating biliary tract cancers: new targets and therapies. Drugs. (2022) 82:1629–47. doi: 10.1007/s40265-022-01808-x 36441502

[10] Zhang Y Zuo C Liu L Hu Y Yang B Qiu S. Single-cell RNA-sequencing atlas reveals an MDK-dependent immunosuppressive environment in ErbB pathway-mutated gallbladder cancer. J Hepatol. (2021) 75:1128–41. doi: 10.1016/j.jhep.2021.06.023 34171432

[11] He H Chen S Yu Y Fan Z Qian Y Dong Y. Comprehensive single-cell analysis deciphered microenvironmental dynamics and immune regulator olfactomedin 4 in pathogenesis of gallbladder cancer. Gut. (2024) 73:1529–42. doi: 10.1136/gutjnl-2023-331773 PMC11347255 38719336

[12] Chancellor D Barrett D Nguyen-Jatkoe L Millington S Eckhardt F. The state of cell and gene therapy in 2023. Mol Ther. (2023) 31:3376–88. doi: 10.1016/j.ymthe.2023.11.001 PMC10727993 37927037

[13] Rouce RH Porteus MH. Cell and gene therapy accessibility. Science. (2024) 385:475. doi: 10.1126/science.ads0252 39088615

[14] Drougkas K Karampinos K Karavolias I Gomatou G Koumprentziotis IA Ploumaki I. CAR-T cell therapy in pancreatic and biliary tract cancers: an updated review of clinical trials. J Gastrointest Cancer. (2024) 55:990–1003. doi: 10.1007/s12029-024-01054-2 38695995

[15] Tang L Pan S Wei X Xu X Wei Q. Arming CAR-T cells with cytokines and more: Innovations in the fourth-generation CAR-T development. Mol Ther. (2023) 31:3146–62. doi: 10.1016/j.ymthe.2023.09.021 PMC10638038 37803832

[16] Myers JA Miller JS. Exploring the NK cell platform for cancer immunotherapy. Nat Rev Clin Oncol. (2021) 18:85–100. doi: 10.1038/s41571-020-0426-7 32934330 PMC8316981

[17] Sabado RL Balan S Bhardwaj N. Dendritic cell-based immunotherapy. Cell Res. (2017) 27:74–95. doi: 10.1038/cr.2016.157 28025976 PMC5223236

[18] Paijens ST Vledder A de Bruyn M Nijman HW. Tumor-infiltrating lymphocytes in the immunotherapy era. Cell Mol Immunol. (2021) 18:842–59. doi: 10.1038/s41423-020-00565-9 PMC8115290 33139907

[19] Wang SW Gao C Zheng YM Yi L Lu JC Huang XY. Current applications and future perspective of CRISPR/Cas9 gene editing in cancer. Mol Cancer. (2022) 21:57. doi: 10.1186/s12943-022-01518-8 35189910 PMC8862238

[20] Xiao Y Yu D. Tumor microenvironment as a therapeutic target in cancer. Pharmacol Ther. (2021) 221:107753. doi: 10.1016/j.pharmthera.2020.107753 33259885 PMC8084948

[21] Mandrekar P Cardinale V. Periostin and mesothelin: Potential predictors of Malignant progression in intrahepatic cholangiocarcinoma. Hepatol Commun. (2018) 2:481–3. doi: 10.1002/hep4.1189 PMC5944583 29761164

[22] Manzanares MA Campbell DJW Maldonado GT Sirica AE. Overexpression of periostin and distinct mesothelin forms predict Malignant progression in a rat cholangiocarcinoma model. Hepatol Commun. (2018) 2:155–72. doi: 10.1002/hep4.1131 PMC5796331 29404524

[23] Wehrli M Guinn S Birocchi F Kuo A Sun Y Larson RC. Mesothelin CAR T cells secreting anti-FAP/anti-CD3 molecules efficiently target pancreatic adenocarcinoma and its stroma. Clin Cancer Res. (2024) 30:1859–77. doi: 10.1158/1078-0432.CCR-23-3841 PMC11062832 38393682

[24] Chen J Hu J Gu L Ji F Zhang F Zhang M. Anti-mesothelin CAR-T immunotherapy in patients with ovarian cancer. Cancer Immunol Immunother. (2023) 72:409–25. doi: 10.1007/s00262-022-03238-w PMC10991348 35925286

[25] Yang Y Vedvyas Y Alcaina Y Trumper SJ Babu DS Min IM. Affinity-tuned mesothelin CAR T cells demonstrate enhanced targeting specificity and reduced off-tumor toxicity. JCI Insight. (2024) 9 (22):e186268. doi: 10.1172/jci.insight.186268 39576012 PMC11601908

[26] Lopez E Hidalgo S Roa E Gomez J Hermansen Truan C Sanders E. Preclinical evaluation of chimeric antigen receptor T cells targeting the carcinoembryonic antigen as a potential immunotherapy for gallbladder cancer. Oncoimmunology. (2023) 12:2225291. doi: 10.1080/2162402X.2023.2225291 37363103 PMC10288912

[27] Zhu HX Yang SH Gao CY Bian ZH Chen XM Huang RR. Targeting pathogenic CD8(+) tissue-resident T cells with chimeric antigen receptor therapy in murine autoimmune cholangitis. Nat Commun. (2024) 15:2936. doi: 10.1038/s41467-024-46654-5 38580644 PMC10997620

[28] Qiao Y Chen J Wang X Yan S Tan J Xia B. Enhancement of CAR-T cell activity against cholangiocarcinoma by simultaneous knockdown of six inhibitory membrane proteins. Cancer Commun (Lond). (2023) 43:788–807. doi: 10.1002/cac2.12452 37282786 PMC10354409

[29] Rizzo A Ricci AD Bonucci C Tober N Palloni A Frega G. Experimental HER2- targeted therapies for biliary tract cancer. Expert Opin Investig Drugs. (2021) 30:389–99. doi: 10.1080/13543784.2021.1854724 33218269

[30] Javle M Borad MJ Azad NS Kurzrock R Abou-Alfa GK George B. Pertuzumab and trastuzumab for HER2-positive, metastatic biliary tract cancer (MyPathway): a multicentre, open-label, phase 2a, multiple basket study. Lancet Oncol. (2021) 22:1290–300. doi: 10.1016/S1470-2045(21)00336-3 34339623

[31] Ohba A Morizane C Ueno M Kobayashi S Kawamoto Y Komatsu Y. Multicenter phase II trial of trastuzumab deruxtecan for HER2-positive unresectable or recurrent biliary tract cancer: HERB trial. Future Oncol. (2022) 18:2351–60. doi: 10.2217/fon-2022-0214 35510484

[32] Harding JJ Fan J Oh DY Choi HJ Kim JW Chang HM. Zanidatamab for HER2-amplified, unresectable, locally advanced or metastatic biliary tract cancer (HERIZON-BTC-01): a multicentre, single-arm, phase 2b study. Lancet Oncol. (2023) 24:772–82. doi: 10.1016/S1470-2045(23)00242-5 37276871

[33] Li X Zhao L Li W Gao P Zhang N. HER2-targeting CAR-T cells show highly efficient anti-tumor activity against glioblastoma both *in vitro* and *in vivo* . Genes Immun. (2024) 25:201–8. doi: 10.1038/s41435-024-00275-6 PMC11178492 38702509

[34] Zhu Y Zhu X Wei X Tang C Zhang W. HER2-targeted therapies in gastric cancer. Biochim Biophys Acta Rev Cancer. (2021) 1876:188549. doi: 10.1016/j.bbcan.2021.188549 33894300

[35] Cutri-French C Nasioudis D George E Tanyi JL. CAR-T cell therapy in ovarian cancer: where are we now? Diagnostics (Basel). (2024) 14 (8):819. doi: 10.3390/diagnostics14080819 38667465 PMC11049291

[36] Mercogliano MF Bruni S Mauro FL Schillaci R. Emerging targeted therapies for HER2-positive breast cancer. Cancers (Basel). (2023) 15 (7):1987. doi: 10.3390/cancers15071987 37046648 PMC10093019

[37] Hegde M Navai S DeRenzo C Joseph SK Sanber K Wu M. Autologous HER2-specific CAR T cells after lymphodepletion for advanced sarcoma: a phase 1 trial. Nat Cancer. (2024) 5:880–94. doi: 10.1038/s43018-024-00749-6 PMC11588040 38658775

[38] Yue S Zhang Y Zhang W. Recent advances in immunotherapy for advanced biliary tract cancer. Curr Treat Options Oncol. (2024) 25:1089–111. doi: 10.1007/s11864-024-01243-y PMC11329538 39066855

[39] Du J Lv X Zhang Z Huang Z Zhang E. Revisiting targeted therapy and immunotherapy for advanced cholangiocarcinoma. Front Immunol. (2023) 14:1142690. doi: 10.3389/fimmu.2023.1142690 36936931 PMC10014562

[40] Gondaliya P Sayyed AA Yan IK Driscoll J Ziemer A Patel T. Targeting PD-L1 in cholangiocarcinoma using nanovesicle-based immunotherapy. Mol Ther. (2024) 32:2762–77. doi: 10.1016/j.ymthe.2024.06.006 PMC11405167 38859589

[41] Schrumpf E Tan C Karlsen TH Sponheim J Bjorkstrom NK Sundnes O. The biliary epithelium presents antigens to and activates natural killer T cells. Hepatology. (2015) 62:1249–59. doi: 10.1002/hep.27840 PMC4589438 25855031

[42] Del Prete A Salvi V Soriani A Laffranchi M Sozio F Bosisio D. Dendritic cell subsets in cancer immunity and tumor antigen sensing. Cell Mol Immunol. (2023) 20:432–47. doi: 10.1038/s41423-023-00990-6 PMC10203372 36949244

[43] Adamik J Munson PV Maurer DM Hartmann FJ Bendall SC Arguello RJ. Immuno-metabolic dendritic cell vaccine signatures associate with overall survival in vaccinated melanoma patients. Nat Commun. (2023) 14:7211. doi: 10.1038/s41467-023-42881-4 37938561 PMC10632482

[44] Han S Lee SY Wang WW Tan YB Sim RHZ Cheong R. A perspective on cell therapy and cancer vaccine in biliary tract cancers (BTCs). Cancers (Basel). (2020) 12 (11):3404. doi: 10.3390/cancers12113404 33212880 PMC7698436

[45] Chouari T La Costa FS Merali N Jessel MD Sivakumar S Annels N. Advances in immunotherapeutics in pancreatic ductal adenocarcinoma. Cancers (Basel). (2023) 15 (17):4265. doi: 10.3390/cancers15174265 37686543 PMC10486452

[46] Takahashi R Yoshitomi M Yutani S Shirahama T Noguchi M Yamada A. Current status of immunotherapy for the treatment of biliary tract cancer. Hum Vaccin Immunother. (2013) 9:1069–72. doi: 10.4161/hv.23844 PMC3899141 23376808

[47] Nakatsuka S Oji Y Horiuchi T Kanda T Kitagawa M Takeuchi T. Immunohistochemical detection of WT1 protein in a variety of cancer cells. Mod Pathol. (2006) 19:804–14. doi: 10.1038/modpathol.3800588 16547468

[48] Marks EI Yee NS. Immunotherapeutic approaches in biliary tract carcinoma: Current status and emerging strategies. World J Gastrointest Oncol. (2015) 7:338–46. doi: 10.4251/wjgo.v7.i11.338 PMC4644856 26600933

[49] Park SY Roh SJ Kim YN Kim SZ Park HS Jang KY. Expression of MUC1, MUC2, MUC5AC and MUC6 in cholangiocarcinoma: prognostic impact. Oncol Rep. (2009) 22:649–57. doi: 10.3892/or_00000485 19639217

[50] Lin B Du L Li H Zhu X Cui L Li X. Tumor-infiltrating lymphocytes: Warriors fight against tumors powerfully. BioMed Pharmacother. (2020) 132:110873. doi: 10.1016/j.biopha.2020.110873 33068926

[51] Alvisi G Termanini A Soldani C Portale F Carriero R Pilipow K. Multimodal single-cell profiling of intrahepatic cholangiocarcinoma defines hyperactivated Tregs as a potential therapeutic target. J Hepatol. (2022) 77:1359–72. doi: 10.1016/j.jhep.2022.05.043 35738508

[52] Liu D Heij LR Czigany Z Dahl E Lang SA Ulmer TF. The role of tumor-infiltrating lymphocytes in cholangiocarcinoma. J Exp Clin Cancer Res. (2022) 41:127. doi: 10.1186/s13046-022-02340-2 35392957 PMC8988317

[53] Bang YH Lee CK Bang K Kim HD Kim KP Jeong JH. Artificial intelligence-powered spatial analysis of tumor-infiltrating lymphocytes as a potential biomarker for immune checkpoint inhibitors in patients with biliary tract cancer. Clin Cancer Res. (2024) 30:4635–43. doi: 10.1158/1078-0432.CCR-24-1265 39150517

[54] Tanaka R Eguchi S Kimura K Ohira G Tanaka S Amano R. Tumor-infiltrating lymphocytes and macrophages as a significant prognostic factor in biliary tract cancer. PloS One. (2023) 18:e0280348. doi: 10.1371/journal.pone.0280348 36693070 PMC9873170

[55] Bo X Wang J Suo T Ni X Liu H Shen S. Tumor-infiltrating mast cells predict prognosis and gemcitabine-based adjuvant chemotherapeutic benefit in biliary tract cancer patients. BMC Cancer. (2018) 18:313. doi: 10.1186/s12885-018-4220-1 29562907 PMC5863450

[56] Kim HD Kim JH Ryu YM Kim D Lee S Shin J. Spatial distribution and prognostic implications of tumor-infiltrating foxP3- CD4+ T cells in biliary tract cancer. Cancer Res Treat. (2021) 53:162–71. doi: 10.4143/crt.2020.704 PMC7812013 32878426

[57] Tanaka R Kimura K Eguchi S Tauchi J Shibutani M Shinkawa H. Preoperative neutrophil-to-lymphocyte ratio predicts tumor-infiltrating CD8(+) T cells in biliary tract cancer. Anticancer Res. (2020) 40:2881–7. doi: 10.21873/anticanres.14264 32366438

[58] Sterner RC Sterner RM. CAR-T cell therapy: current limitations and potential strategies. Blood Cancer J. (2021) 11:69. doi: 10.1038/s41408-021-00459-7 33824268 PMC8024391

[59] Creelan BC Wang C Teer JK Toloza EM Yao J Kim S. Tumor-infiltrating lymphocyte treatment for anti-PD-1-resistant metastatic lung cancer: a phase 1 trial. Nat Med. (2021) 27:1410–8. doi: 10.1038/s41591-021-01462-y PMC8509078 34385708

[60] Gachechiladze M Skarda J Skanderova D Uberall I Kolek V Smickova P. Prognostic value of tumor-infiltrating lymphocytes (TILs) and their association with PD-L1 expression and DNA repair protein RAD51 in patients with resected non-small cell lung carcinoma. Lung Cancer. (2020) 147:30–8. doi: 10.1016/j.lungcan.2020.06.025 32653671

[61] Fontugne J Augustin J Pujals A Compagnon P Rousseau B Luciani A. PD-L1 expression in perihilar and intrahepatic cholangiocarcinoma. Oncotarget. (2017) 8:24644–51. doi: 10.18632/oncotarget.15602 PMC5421876 28445951

[62] Ye Y Zhou L Xie X Jiang G Xie H Zheng S. Interaction of B7-H1 on intrahepatic cholangiocarcinoma cells with PD-1 on tumor-infiltrating T cells as a mechanism of immune evasion. J Surg Oncol. (2009) 100:500–4. doi: 10.1002/jso.21376 19697355

[63] Lim YJ Koh J Kim K Chie EK Kim S Lee KB. Clinical implications of cytotoxic T lymphocyte antigen-4 expression on tumor cells and tumor-infiltrating lymphocytes in extrahepatic bile duct cancer patients undergoing surgery plus adjuvant chemoradiotherapy. Target Oncol. (2017) 12:211–8. doi: 10.1007/s11523-016-0474-1 28084572

[64] Ueno T Tsuchikawa T Hatanaka KC Hatanaka Y Mitsuhashi T Nakanishi Y. Prognostic impact of programmed cell death ligand 1 (PD-L1) expression and its association with epithelial-mesenchymal transition in extrahepatic cholangiocarcinoma. Oncotarget. (2018) 9:20034–47. doi: 10.18632/oncotarget.25050 PMC5929444 29732001

[65] Nakamura H Arai Y Totoki Y Shirota T Elzawahry A Kato M. Genomic spectra of biliary tract cancer. Nat Genet. (2015) 47:1003–10. doi: 10.1038/ng.3375 26258846

[66] Liu Y Huang W Cai Z. Synthesizing AND gate minigene circuits based on CRISPReader for identification of bladder cancer cells. Nat Commun. (2020) 11:5486. doi: 10.1038/s41467-020-19314-7 33127914 PMC7599332

[67] Nissim L Wu MR Pery E Binder-Nissim A Suzuki HI Stupp D. Synthetic RNA-based immunomodulatory gene circuits for cancer immunotherapy. Cell. (2017) 171:1138–50:e1115. doi: 10.1016/j.cell.2017.09.049 PMC5986174 29056342

[68] Roybal KT Rupp LJ Morsut L Walker WJ McNally KA Park JS. Precision tumor recognition by T cells with combinatorial antigen-sensing circuits. Cell. (2016) 164:770–9. doi: 10.1016/j.cell.2016.01.011 PMC4752902 26830879

[69] Williams JZ Allen GM Shah D Sterin IS Kim KH Garcia VP. Precise T cell recognition programs designed by transcriptionally linking multiple receptors. Science. (2020) 370:1099–104. doi: 10.1126/science.abc6270 PMC8054651 33243890

[70] Frankel NW Deng H Yucel G Gainer M Leemans N Lam A. Precision off-the-shelf natural killer cell therapies for oncology with logic-gated gene circuits. Cell Rep. (2024) 43:114145. doi: 10.1016/j.celrep.2024.114145 38669141

[71] Chen J Amoozgar Z Liu X Aoki S Liu Z Shin SM. Reprogramming the intrahepatic cholangiocarcinoma immune microenvironment by chemotherapy and CTLA-4 blockade enhances anti-PD-1 therapy. Cancer Immunol Res. (2024) 12:400–12. doi: 10.1158/2326-6066.CIR-23-0486 PMC10985468 38260999

[72] Biffi G Tuveson DA. Diversity and biology of cancer-associated fibroblasts. Physiol Rev. (2021) 101:147–76. doi: 10.1152/physrev.00048.2019 PMC7864232 32466724

[73] Ilyas SI Affo S Goyal L Lamarca A Sapisochin G Yang JD. Cholangiocarcinoma - novel biological insights and therapeutic strategies. Nat Rev Clin Oncol. (2023) 20:470–86. doi: 10.1038/s41571-023-00770-1 PMC10601496 37188899

[74] Chen Y McAndrews KM Kalluri R. Clinical and therapeutic relevance of cancer-associated fibroblasts. Nat Rev Clin Oncol. (2021) 18:792–804. doi: 10.1038/s41571-021-00546-5 34489603 PMC8791784

[75] Cantallops Vila P Ravichandra A Agirre Lizaso A Perugorria MJ Affo S. Heterogeneity, crosstalk, and targeting of cancer-associated fibroblasts in cholangiocarcinoma. Hepatology. (2024) 79:941–58. doi: 10.1097/HEP.0000000000000206 37018128

[76] Affo S Nair A Brundu F Ravichandra A Bhattacharjee S Matsuda M. Promotion of cholangiocarcinoma growth by diverse cancer-associated fibroblast subpopulations. Cancer Cell. (2021) 39:866–882.e811. doi: 10.1016/j.ccell.2021.03.012 33930309 PMC8241235

[77] Xu M Zhang T Xia R Wei Y Wei X. Targeting the tumor stroma for cancer therapy. Mol Cancer. (2022) 21:208. doi: 10.1186/s12943-022-01670-1 36324128 PMC9628074

[78] Zhao J Chen J Li C Xiang H Miao X. Hyaluronidase overcomes the extracellular matrix barrier to enhance local drug delivery. Eur J Pharm Biopharm. (2024) 203:114474. doi: 10.1016/j.ejpb.2024.114474 39191305

[79] Hosein AN Brekken RA Maitra A. Pancreatic cancer stroma: an update on therapeutic targeting strategies. Nat Rev Gastroenterol Hepatol. (2020) 17:487–505. doi: 10.1038/s41575-020-0300-1 32393771 PMC8284850

[80] Ettrich TJ Ebert M Lorenzen S Moehler M Vogel A Witkowski L. ASCO- and ESMO-update 2017 - highlights of the 53. meeting of the American Society of Clinical Oncology/ASCO 2017 and European Society for Medical Oncology/ESMO congress 2017. Z Gastroenterol. (2018) 56:384–97. doi: 10.1055/s-0044-101757 29642252

[81] Van Cutsem E Tempero MA Sigal D Oh DY Fazio N Macarulla T. Randomized phase III trial of pegvorhyaluronidase alfa with nab-paclitaxel plus gemcitabine for patients with hyaluronan-high metastatic pancreatic adenocarcinoma. J Clin Oncol. (2020) 38:3185–94. doi: 10.1200/JCO.20.00590 PMC7499614 32706635

[82] Wang S Li Y Xu C Dong J Wei J. An oncolytic vaccinia virus encoding hyaluronidase reshapes the extracellular matrix to enhance cancer chemotherapy and immunotherapy. J Immunother Cancer. (2024) 12 (3):e008431. doi: 10.1136/jitc-2023-008431 38458640 PMC10921532

[83] Isidan A Yenigun A Soma D Aksu E Lopez K Park Y. Development and characterization of human primary cholangiocarcinoma cell lines. Am J Pathol. (2022) 192:1200–17. doi: 10.1016/j.ajpath.2022.05.007 PMC9472155 35640676

[84] Pan S Hu Y Gan L Lai J Zheng P Zhang Y. Matrix metalloproteinase-2 inducing COL1A1 synthesis via integrin alpha V promotes invasion and metastasis of cholangiocarcinoma cells. Ann Hepatol. (2024) 29:101279. doi: 10.1016/j.aohep.2023.101279 38123132

[85] Mancarella S Serino G Dituri F Cigliano A Ribback S Wang J. Crenigacestat, a selective NOTCH1 inhibitor, reduces intrahepatic cholangiocarcinoma progression by blocking VEGFA/DLL4/MMP13 axis. Cell Death Differ. (2020) 27:2330–43. doi: 10.1038/s41418-020-0505-4 PMC7370218 32042099

[86] Song X Si Q Qi R Liu W Li M Guo M. Indoleamine 2,3-dioxygenase 1: A promising therapeutic target in Malignant tumor. Front Immunol. (2021) 12:800630. doi: 10.3389/fimmu.2021.800630 35003126 PMC8733291

[87] Tang K Wu YH Song Y Yu B. Indoleamine 2,3-dioxygenase 1 (IDO1) inhibitors in clinical trials for cancer immunotherapy. J Hematol Oncol. (2021) 14:68. doi: 10.1186/s13045-021-01080-8 33883013 PMC8061021

[88] Sun C Wang B Hao S. Adenosine-A2A receptor pathway in cancer immunotherapy. Front Immunol. (2022) 13:837230. doi: 10.3389/fimmu.2022.837230 35386701 PMC8977492

[89] Martinez-Ordonez A Duran A Ruiz-Martinez M Cid-Diaz T Zhang X Han Q. Hyaluronan driven by epithelial aPKC deficiency remodels the microenvironment and creates a vulnerability in mesenchymal colorectal cancer. Cancer Cell. (2023) 41:252–271.e259. doi: 10.1016/j.ccell.2022.11.016 36525970 PMC9931663

[90] Lee YE Go GY Koh EY Yoon HN Seo M Hong SM. Synergistic therapeutic combination with a CAF inhibitor enhances CAR-NK-mediated cytotoxicity via reduction of CAF-released IL-6. J Immunother Cancer. (2023) 11 (2):e006130. doi: 10.1136/jitc-2022-006130 36849201 PMC9972461

[91] Zheng R Shen K Liang S Lyu Y Zhang S Dong H. Specific ECM degradation potentiates the antitumor activity of CAR-T cells in solid tumors. Cell Mol Immunol. (2024) 21:1491–504. doi: 10.1038/s41423-024-01228-9 PMC11606952 39472748

[92] Waldhauer I Gonzalez-Nicolini V Freimoser-Grundschober A Nayak TK Fahrni L Hosse RJ. Simlukafusp alfa (FAP-IL2v) immunocytokine is a versatile combination partner for cancer immunotherapy. MAbs. (2021) 13:1913791. doi: 10.1080/19420862.2021.1913791 33974508 PMC8115765

